# The longitudinal association between possible new sarcopenia and the depression trajectory of individuals and their intimate partners

**DOI:** 10.3389/fnagi.2022.1001241

**Published:** 2022-12-05

**Authors:** Yufeng Tian, Zhigang Hu, Xinyu Song, Ailan Yang

**Affiliations:** ^1^Department of Evidence Based Medicine Center, The First College of Clinical Medicine Science, China Three Gorges University, Yichang, China; ^2^Department of Academic Management, Clinical Research Center, China Three Gorges University, Yichang, China; ^3^Department of Respiratory and Critical Care Medicine, Yichang Central People's Hospital at Zhijiang, Zhijiang, China; ^4^Department of Respiratory and Critical Care Medicine, Yichang Central People's Hospital, Yichang, China

**Keywords:** depression, intimate partners, trajectory, sex difference, possible sarcopenia

## Abstract

**Background:**

It is currently unknown whether the dynamic nature of depression affects the development of sarcopenia. Herein, this study aims to assess the association between possible new sarcopenia and the depression trajectory of individuals and their intimate partners through a 4-year longitudinal cohort study.

**Methods:**

Our study included 784 pairs of individuals without possible sarcopenia and their spouses from the China Health and Retirement Longitudinal Study (CHARLS) 2011. All individuals and their spouses received three assessments of the Center for Epidemiologic Studies Depression 10-item (CESD-10) scale in 2011, 2013, and 2015. According to the diagnostic algorithm recommended by the Asian Working Group for Sarcopenia (AWGS) 2019, we evaluated the incidence of possible sarcopenia in individuals in 2015. Latent class analysis (LCA) was used to identify a longitudinal depression trajectory of individuals and their spouses during a 4-year follow-up. Subsequently, we assessed the relationship between possible sarcopenia and depression trajectory using three generalized additive models.

**Results:**

In 2015, 24.87% (195/784) of individuals were diagnosed with possible sarcopenia. LCA identified five depression trajectories: a persistently high risk of depression in individuals and their spouses (reference; class 1 = 34 [4.3%]); a persistently low risk of depression in individuals and their spouses (class 2 = 526 [67.1%]); a high risk of depression in individuals and a low risk of depression in spouses (class 3 = 46 [5.9%]); a low risk of depression in individuals and a high risk of depression in spouses (class 4 = 116 [14.8%]); and a reduced risk of depression in individuals and their spouses (class 5 = 62 [7.9%]). The highest incidence of possible sarcopenia was shown in class 1, followed by classes 3 and 5. Classes 2 (adjusted relative risk (RR) = 0.44, 95% confidence interval (CI): 0.20–0.97) and 4 (adjusted RR = 0.40, 95%CI: 0.17–0.96) had a significantly lower incidence of possible sarcopenia than class 1. Subgroup analysis demonstrated that the incidence of possible sarcopenia in class 4 was obviously higher in women (38.89%) than in men (18.4%).

**Conclusions:**

Our study indicates a persistently high risk of depression in individuals to develop possible sarcopenia. In addition, a persistently high risk of depression in intimate partners potentially increases the risk of possible new sarcopenia, especially in female individuals who are at low risk of depression.

## Introduction

Population aging has become a serious and rapidly growing problem in the world, especially in China. The proportion of older people in China continued to show an upward trend from 1960 to 2016, while the proportion of young people and the birth rates demonstrated a downward trend (Wei et al., 2019). Age-related loss of skeletal muscle, named “sarcopenia,” can lead to physiological consequences and adverse clinical outcomes (Cruz-Jentoft and Sayer, 2019). In 2019, the Asian Working Group for Sarcopenia (AWGS) proposed the following original definition of sarcopenia: age-related loss of skeletal muscle mass plus loss of muscle strength and/or reduced physical performance (Chen L. K. et al., 2020). The AWGS 2019 also introduced the conception of “possible sarcopenia” to encourage older people to attend hospital for a confirmatory diagnosis and receive early lifestyle interventions for possible sarcopenia. Clinicians are exempted from actively exploring potential causes, especially reversible ones, and providing appropriate personalized intervention countermeasures for older people with possible sarcopenia (Chen L. K. et al., 2020). Current studies have also suggested that possible sarcopenia is associated with decreased physical function (Kristensen et al., 2021; Lim and Kong, 2022) and increased risks of cognitive impairment (Maeda and Akagi, 2017; Cipolli et al., 2021), stroke (6), and 1-year mortality (Kristensen et al., 2021).

Depression is a common and important health problem in the elderly population (Delibaş et al., 2021). Approximately one in five people is likely to experience depression at some point in their lifetime (Malhi and Mann, 2018). Reciprocal associations between depression and age-related diseases have generated pathogenetic hypotheses and provided targets for treatment development (Alexopoulos, 2019). Geriatric depression is positively associated with adverse clinical consequences, which include medical comorbidities, cognitive impairment, poor functioning, and overall mortality (Valiengo et al., 2016). However, the association between depression and sarcopenia is controversial. A cross-sectional study from Brazil reported that depression was associated with an increased risk of sarcopenia (odds ratio (OR) = 2.23, 95% confidence interval [CI] = 1.11–4.48) but not with pre-sarcopenia (Szlejf et al., 2019). Endo et al. (2021) found that depression was positively associated with pre-sarcopenia and sarcopenia. A Korean study observed non-significant associations between the prevalence of sarcopenia and depression or depressive symptoms (Byeon et al., 2016). Overall, most studies were cross-sectional, which limited the ability to establish a causal relationship between depression and sarcopenia. The course of depression varies considerably over a lifetime. After treatment, depression may recover, recur, and persist. Compared to acute depression and controls, chronic depression was associated with an increased risk of developing adverse consequences (Kahl et al., 2017). However, no study investigated the association between the dynamic nature of depression and sarcopenia. In addition, recent studies have found that depression in intimate partners may have negative effects on the mental health of individuals and health-related quality of life (Li et al., 2016; Franz et al., 2020). Whether depression in intimate partners affects possible new sarcopenia in individuals remains to be studied further.

Therefore, we hypothesized that different depression trajectories in individuals and intimate partners may be associated with the different incidences of sarcopenia. We obtained study population data from the China Health and Retirement Longitudinal Study (CHARLS), and determined a longitudinal depression trajectory of individuals and their spouses using latent class analysis (LCA). Further analyses were used to assess the relationships between different depression trajectories and sarcopenia and explore the sex difference of these relationships.

## Methods

### Study population

The CHARLS is a nationally representative longitudinal survey to better understand the socioeconomic determinants and consequences of aging for individuals aged ≥ 45 years and their intimate partners. The CHARLS completed the first widespread survey during June 2011 and March 2012, which included 17,708 individuals from 150 county-level units within 28 provinces. Face-to-face computer-assisted personal interviews and physical measurements were conducted at each 2-year follow-up. To facilitate comparisons with the RAND HRS data, researchers have created the harmonized CHARLS data. The Biomedical Ethics Review Committee of Peking University approved the CHARLS. Written informed consents and all survey data were collected in the National School of Development of Peking University. A more detailed description of the CHARLS has been reported elsewhere (Zhao et al., 2014) and is given in the following link: http://charls.pku.edu.cn/en/.

### The definition of sarcopenia and depression

The CHARLS had no data on appendicular skeletal muscle mass measured by dual-energy X-ray absorptiometry (DXA) and bioelectrical impedance analysis (BIA). Therefore, we assessed the effect of depression on possible sarcopenia. According to the AWGS 2019, possible sarcopenia was defined as low muscle strength with or without reduced physical performance (Chen L. K. et al., 2020). Handgrip strength <28.0 kg for men and <18.0 kg for women were regarded as low muscle strength. The five-time chair stand test ≥12 s was considered as low physical performance. The age cutoffs for possible sarcopenia were set at 60 years. In 2011, all individuals with possible sarcopenia were excluded from this study. In 2015, all included individuals in our study population received reassessment for possible sarcopenia.

Researchers of the CHARLS measured depressive symptoms using the 10-item Center for Epidemiologic Studies Depression (CES-D) scale. The study by Chen et al. confirmed adequate reliability and validity for assessing depression in the elderly Chinese community-dwelling population (Cheng et al., 2016). Each CES-D item had four answers, including “rarely,” “some days” (1–2 days), “occasionally” (3–4 days), and “most of the time” (5–7 days). We collected all answers as values, from 0 to 3 as “rarely” and “most of the time.” Total scores of 10 items were 30 scores and the CES-D ≥ 12 scores were classified as depression with reference to previous studies (Cheng et al., 2016; Ruiz et al., 2019; Chen H. et al., 2020; Chen L. K. et al., 2020). Individuals and their intimate partners in our study must have detailed data on depression in 2011, 2013, and 2015. Intimate partners were rationed to spouses.

### Variables

According to previous studies (Kim et al., 2015; Jiang et al., 2020; Hu Y. et al., 2022; Hu Z. et al., 2022; Gao K. et al., 2021), we collected some potentially confounding variables to adjust for the association between depression trajectory and possible sarcopenia. These variables included demographic characteristics (sex, age, urban/rural, education levels, and body mass index), physical/behavioral factors (smoking, drinking alcohol, and difficulty scores of mobility activities), and 13 physician-diagnosed comorbidities (hypertension, dyslipidemia, hyperglycemia, cancers, chronic lung disease, liver disease, heart disease, stroke, kidney disease, digestive disease, emotional or nervous problems, arthritis, and asthma).

Following our previous study (Hu et al., 2021), body mass index was categorized into four groups: underweight (<18.5 kg/m^2^), normal (18.5 to <24.0 kg/m^2^), overweight (24.0 to <28.0 kg/m^2^), and obese (≥28.0 kg/m^2^). The status of smoking and drinking alcohol was divided into never, ever, and current. In addition, we also obtained the difficulty scores of mobility activities. The scores of mobility activities on the CHARLS summarized 7-item scores of having any difficulty (yes = 1 score), which included walking 100 m, climbing several flights of stairs, getting up from a chair, stooping or kneeling or crouching, extending arms up, lifting 5 kg, and picking up a small coin. Mobility difficulties were found to be a mediator of correlations between depression and chronic diseases (Jiang et al., 2020).

### Inclusion and exclusion criteria

All included individuals must meet the following criteria: (1) the age of the included individuals was more than 60 years; (2) individuals and their spouses had three CEDS-10 assessments in 2011, 2013, and 2015; and (3) individuals experienced two anthropometric and physical measurements in 2011 and 2015. We also excluded the following individuals in this study: (1) individuals were diagnosed with possible sarcopenia in the CHARLS 2011 and (2) individuals did not have data for the confounding factors included in the CHARLS 2011.

### Statistical analysis

Our statistical analyses had three components. Firstly, the study population was stratified according to the occurrence of possible sarcopenia. Using SPSS, the Chi-squared test was used to compare the difference between the two groups with categorical variables presented as counts and percentages (%). We exhibited continuous variables as means and standard deviations (SDs), and performed a comparison of the two groups using the Mann–Whitney U test for skewed continuous variables and Student's *t*-test or one-way analysis of variance (ANOVA) for normally distributed continuous variables. Secondly, latent class analysis (LCA) was used to determine a class-based phenotype with respect to the longitudinal depression in individuals and their spouses within our study population. The LCA model might identify solutions that best describe these latent classes within which the indicators follow the same distribution (Sinha et al., 2021). Once identified mathematically, the latent classes are internally homogeneous, but distinct from each other (Sinha et al., 2021). We determined the best fit of the latent classes based on multiple indices, which included the Akaike information criterion (AIC), Bayesian information criterion (BIC), adjusted BIC (aBIC), entropy, Lo-Mendell-Rubin test (LMRt), and bootstrap likelihood ratio test (BLRt). Lower values of AIC, BIC, and aBIC with the highest value of entropy indicated a better fit. In addition, the best fit of the latent classes was associated with LMRt and BLRt < 0.05. Thirdly, generalized additive analyses with three binomial regression models estimated the association between possible new-onset sarcopenia with a longitudinal depression trajectory. Model 1 included demographic characteristics (sex, age, urban/rural, education levels, and body mass index), model 2 added behavioral factors (smoking, drinking alcohol, and difficulty scores of mobility activities), and model 3 added behavioral factors and the 13 abovementioned comorbidities. The first and third part of statistical analyses were done in Empower(R) (www.empowerstats.com; X&Y solutions, Inc., Boston, MA, USA). Mplus completed LCA. Relative risks (RRs) with 95% CI represented the strength of all associations, and a two-tailed *p* < 0.05 was considered statistically significant.

### Ethics

Because all related data were derived from the open CHALRS, no patients were involved in the recruitment and conduct of the study. This study was deemed exempt from review by the Institutional Review Board.

## Results

### Study population characteristics

**Figure 1:**
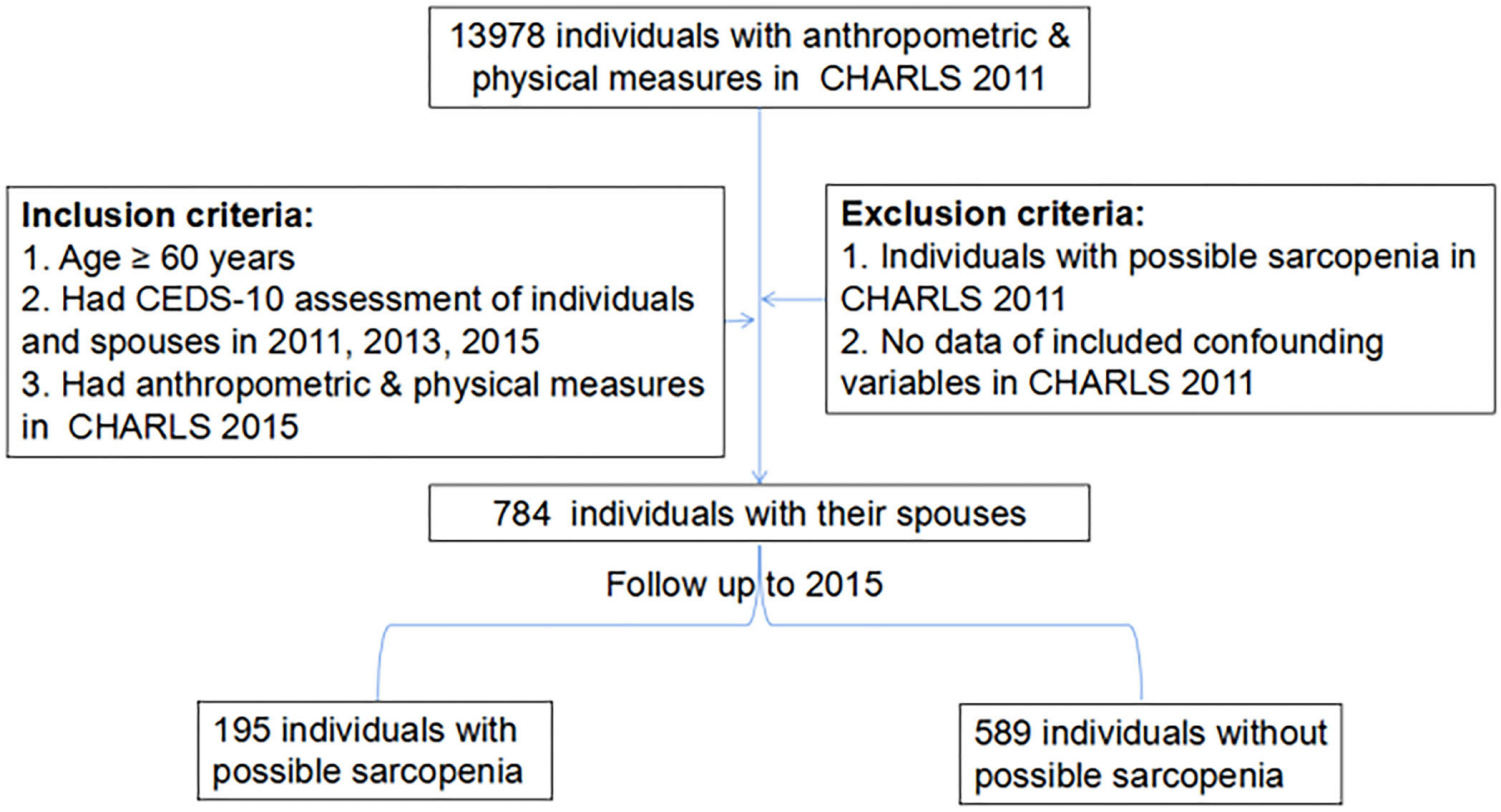
Flow diagram of the study population. The study population was grouped according to whether individuals had possible sarcopenia in the China Health and Retirement Longitudinal Study 2015.

A total of 784 individuals along with their spouses in the CHARLS 2011 were included in this study. A flow diagram is shown in Figure 1. Among the included patients with an average age of 64.8 ± 4.2 years, 64% were men, and 65.3% were from rural areas. Approximately 51.9 and 52.3% of individuals had no history of smoking and drinking alcohol, respectively. Approximately 9.4% of individuals were associated with the difficulty scores of mobility activities ≥1 score. In our study, the prevalence of depression in an individual was 18.9% in 2011, 14.8% in 2013, and 16.6% in 2015. The proportion of an individual's intimate partners with depression was 24.5% in 2011, 19% in 2013, and 23.3% in 2015. A total of 195 individuals met the diagnostic criterion for possible sarcopenia in the CHARLS 2015. Individuals with possible sarcopenia were associated with older age, higher difficulty scores in mobility activities, and a higher prevalence of depression than those without possible sarcopenia. Detailed characteristics between individuals with and without possible sarcopenia are shown in Table 1.

**Table 1:** Study population characteristics in the China health and retirement longitudinal study.

	**Individuals with possible sarcopenia**		***P*-value**
	**No**	**Yes**	
N	589	195	
**Age**			<0.001
60 to 69 y	526 (89.3%)	152 (77.9%)	
≥70 y	63 (10.7%)	43 (22.1%)	
**Sex**			0.403
Male	382 (64.9%)	120 (61.5%)	
Female	207 (35.1%)	75 (38.5%)	
**Education levels**			0.18
Under elementary school	216 (36.7%)	86 (44.1%)	
Elementary and middle school	320 (54.3%)	93 (47.7%)	
High school or above	53 (9.0%)	16 (8.2%)	
**Urban/rural**			<0.001
Urban	225 (38.2%)	47 (24.1%)	
Rural	364 (61.8%)	148 (75.9%)	
**Body mass index (BMI)**			0.008
Underweight (<18.5 kg/m2)	27 (4.6%)	21 (10.8%)	
Normal weight (18.5 to 24.9 kg/m2)	316 (53.7%)	101 (51.8%)	
Overweight (25 to 27.9 kg/m2)	201 (34.1%)	54 (27.7%)	
Obesity (> 28 kg/m2)	45 (7.6%)	19 (9.7%)	
**Drinking alcohol**			0.314
Never	295 (50.1%)	102 (52.3%)	
Ever	64 (10.9%)	27 (13.8%)	
Current	230 (39.0%)	66 (33.8%)	
**Smoking**			0.445
Never	300 (50.9%)	107 (54.9%)	
Ever	69 (11.7%)	25 (12.8%)	
Current	220 (37.4%)	63 (32.3%)	
Difficulty scores of mobility activities	0.77 ± 1.06	1.55 ± 1.27	0.002
**Hypertension**			0.41
No	420 (71.3%)	133 (68.2%)	
Yes	169 (28.7%)	62 (31.8%)	
**Diabetes**			0.324
No	550 (93.4%)	178 (91.3%)	
Yes	39 (6.6%)	17 (8.7%)	
**Dyslipidemia**			0.873
No	522 (88.6%)	172 (88.2%)	
Yes	67 (11.4%)	23 (11.8%)	
**Cancers**			0.706
No	582 (98.8%)	192 (98.5%)	
Yes	7 (1.2%)	3 (1.5%)	
**Chronic lung diseases**			0.068
No	527 (89.5%)	165 (84.6%)	
Yes	62 (10.5%)	30 (15.4%)	
**Heart disease**			0.693
No	511 (86.8%)	167 (85.6%)	
Yes	78 (13.2%)	28 (14.4%)	
**Stroke**			0.773
No	576 (97.8%)	190 (97.4%)	
Yes	13 (2.2%)	5 (2.6%)	
**Emotional or nervous problems**			0.432
No	586 (99.5%)	193 (99.0%)	
Yes	3 (0.5%)	2 (1.0%)	
**Arthritis**			0.044
No	417 (70.8%)	123 (63.1%)	
Yes	172 (29.2%)	72 (36.9%)	
**Liver diseases**			0.61
No	573 (97.3%)	191 (97.9%)	
Yes	16 (2.7%)	4 (2.1%)	
**Kidney diseases**			0.349
No	560 (95.1%)	182 (93.3%)	
Yes	29 (4.9%)	13 (6.7%)	
**Digestive diseases**			0.178
No	468 (79.5%)	146 (74.9%)	
Yes	121 (20.5%)	49 (25.1%)	
**Asthma**			0.081
No	558 (94.7%)	178 (91.3%)	
Yes	31 (5.3%)	17 (8.7%)	
**Depression of individuals in 2011**			0.018
No	489 (83.0%)	147 (75.4%)	
Yes	100 (17.0%)	48 (24.6%)	
**Depression of individuals in 2013**			0.033
No	511 (86.8%)	157 (80.5%)	
Yes	78 (13.2%)	38 (19.5%)	
**Depression of individuals in 2015**			<0.001
No	507 (86.1%)	145 (74.4%)	
Yes	82 (13.9%)	50 (25.6%)	
**Depression of individuals spouse in 2011**		0.23	0.23
No	451 (76.6%)	141 (72.3%)	
Yes	138 (23.4%)	54 (27.7%)	
**Depression of individuals' spouse in 2013**		0.407	0.407
No	481 (81.7%)	154 (79.0%)	
Yes	108 (18.3%)	41 (21.0%)	
**Depression of individuals' spouse in 2015**		0.041	0.041
No	462 (78.4%)	139 (71.3%)	
Yes	127 (21.6%)	56 (28.7%)	

### Latent class analysis

A five-class model had a higher entropy (0.817) and a lower AIC and aBIC than two to four classes in our study population. When the class model was divided into six groups, LMRt and BLRt were more than 0.05 with increased aBIC (see Table 2). Overall, the five-class model was chosen as the best-fitting model solution for a longitudinal depression trajectory of individuals and their spouses.

**Table 2:** Fit indices for latent class analysis (LCA) models with 2–8 classes.

	**AIC**	**BIC**	**aBIC**	**Entropy**	**LMRt**	**BLRt**
Class 2	4278.287	4338.924	4297.643	0.680	<0.01	<0.01
Class 3	4179.337	4272.625	4209.115	0.783	<0.01	<0.01
Class 4	4153.423	4279.362	4193.623	0.752	0.096	<0.01
Class 5	4144.170	4302.760	4192.792	0.817	<0.05	<0.01
Class 6	4140.030	4331.270	4201.075	0.831	0.066	0.182
Class 7	4147.691	4371.582	4219.158	0.849	0.148	0.600
Class 8	4154.910	4411.452	4236.799	0.793	0.456	0.500

AIC, Akaike's information criterion; BIC, Bayesian information criterion; aBIC, adjusted Bayesian information criterion; LMRt, Lo-Mendell-Rubin test; BLRt, Bootstrap likelihood ratio test.

**Figure 2:**
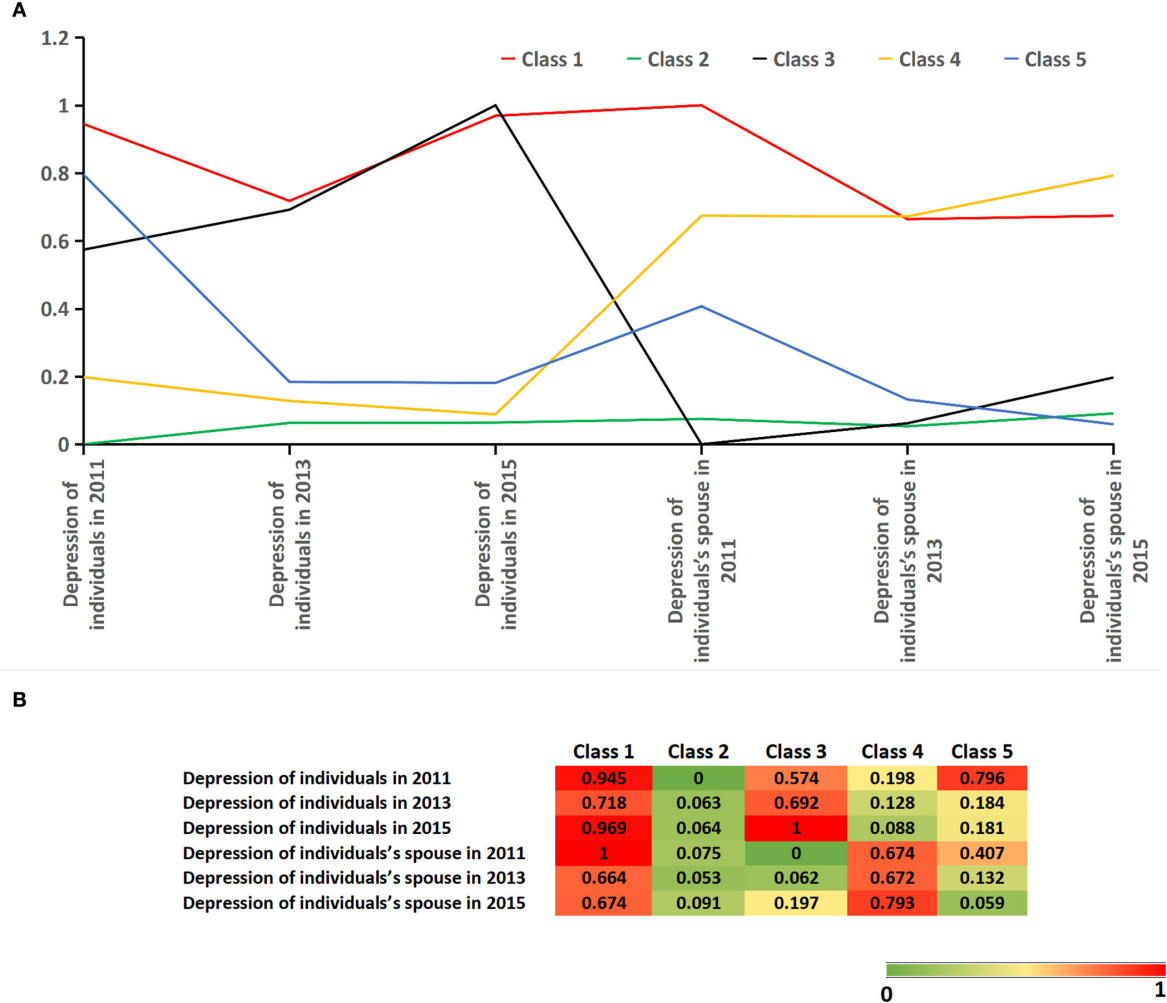
**(A)** Latent class analysis (LCA) of depression trajectories in individuals and their spouses. Class 1: a persistently high risk of depression in individuals and their spouses (reference; *n* = 34 [4.3%]); class 2: a persistently low risk of depression in individuals and their spouses (*n* = 526 [67.1%]); class 3: a high risk of depression in individuals and a low risk of depression in individuals' spouses (*n* = 46 [5.9%]); class 4: a low risk of depression in individuals and a high risk of depression in individuals' spouses (*n* = 116 [14.8%]; and class 5: the decreased risk of depression in individuals and their spouses (*n* = 62 [7.9%]. **(B)** Probability of each indicator variable in five depression trajectories.

Figure 2 displays five depression trajectories and the probability of latent class membership for each indicator variable. Class 1 represented a persistently high risk of depression in individuals and their spouses (reference; *n* = 34 [4.3%]). Class 2 was characterized by a persistently low risk of depression in individuals and their spouses (*n* = 526 [67.1%]). Class 3 had a high risk of depression in individuals and a low risk of depression in individuals' spouses (*n* = 46 [5.9%]). Class 4 contained a low risk of depression in individuals and a high risk of depression in individuals' spouses (*n* = 116 [14.8%]). Class 5 harbored the lowest risk of depression in individuals and their spouses (*n* = 62 [7.9%]).

Class 4 might be used to assess the effect of depression in individuals' spouses on possible sarcopenia in individuals without depression. The main difference between classes 1 and 3 was that individual's spouses were at persistently high risk of depression in class 1. Individuals who were at a persistently high risk of depression were regarded to be chronically depressed. Individuals in class 5 might be considered depression remission or acute depression.

### The association between a longitudinal depression trajectory and possible sarcopenia

**Figure 3:**
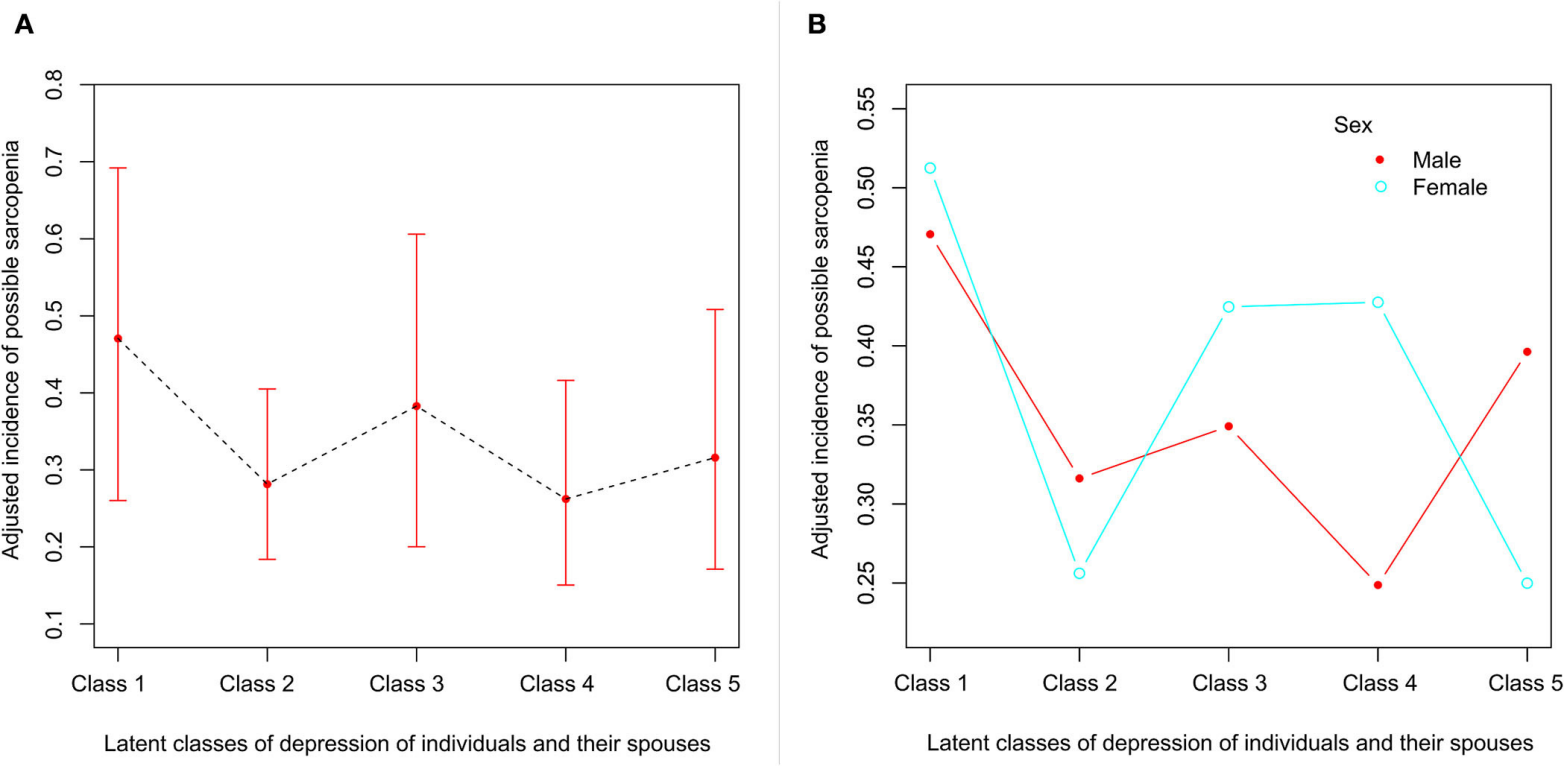
**(A)** The adjusted incidence of the different latent classes of depression in individuals and their spouses. Class 1: a persistently high risk of depression in individuals and their spouses; class 2: a persistently low risk of depression in individuals and their spouses; class 3: a high risk of depression in individuals and a low risk of depression in individuals' spouses; class 4: a low risk of depression in individuals and a high risk of depression in individuals' spouses; and class 5: the decreased risk of depression in individuals and their spouses. **(B)** The adjusted incidence of the different latent classes of depression in individuals and their spouses when stratified by sex.

The highest incidence of developing possible sarcopenia was shown in class 1 (47.1%), followed by classes 3 (37%), 5 (32.3%), 2 (22.2%), and 4 (21.6%). Figure 3A demonstrates the incidence of possible sarcopenia in class 5 after adjusting for demographic characteristics, behavioral factors, and comorbidities. All three models suggested that there was a significantly lower incidence of possible sarcopenia in classes 2 (adjusted RR = 0.44, 95%CI: 0.20–0.97 in model 3) and 4 (adjusted RR = 0.40, 95%CI: 0.17–0.96 in model 3) than in class 1. The risks of developing possible sarcopenia in classes 3 and 5 demonstrated a downward trend compared with class 1, although the difference did not reach statistical significance (see Table 3). Interestingly, the sex difference was shown in classes 4 and 5 (see Figure 3B). When individuals were women who were at persistently low risk of depression, a persistently high risk of depression in their spouses was associated with an increased risk of developing possible sarcopenia class 4. However, male individuals were not affected by their spouses in the incidence of possible sarcopenia. The incidence of possible sarcopenia in women with a decreased risk of depression (class 5) was similar to that of those who were at persistently low risk of depression (class 2), while an upward trend of developing possible sarcopenia in man was shown in class 5 compared with class 2.

**Table 3:** Associations between possible sarcopenia and depression in individuals and their spouses.

	**Model 1**	**Model 2**	**Model 3**
Class 1	Ref	Ref	Ref
Class 2	0.32 (0.15, 0.67)^‡^	0.40 (0.18, 0.86)^†^	0.44 (0.20, 0.97)^†^
Class 3	0.66 (0.26, 1.65)	0.65 (0.25, 1.69)	0.70 (0.26, 1.86)
Class 4	0.32 (0.14, 0.73)^‡^	0.38 (0.16, 0.89)^†^	0.40 (0.17, 0.96)^†^
Class 5	0.47 (0.19, 1.16)	0.50 (0.20, 1.23)	0.52 (0.21, 1.31)

Model 1 adjusted the following variables: sex, age, urban/rural, education levels, and body mass index.

Model 2 adjusted the following variables: sex, age, urban/rural, education levels, body mass index, smoking, drinking alcohol, and difficulty scores of mobility activities.

Model 3 adjusted the following variables: sex, age, urban/rural, education levels, body mass index, smoking, drinking alcohol, difficulty scores of mobility activities, hypertension, dyslipidemia, hyperglycemia, cancers, chronic lung diseases, liver diseases, heart diseases, stroke, kidney diseases, digestive diseases, emotional or nervous problems, arthritis, and asthma.

Class 1: persistently high risk of depression in individuals and their spouses.

Class 2: persistently low risk of depression in individuals and their spouses.

Class 3: high risk of depression in individuals and low risk of depression in individuals' spouses.

Class 4: low risk of depression in individuals and high risk of depression in individual's spouses.

Class 5: decreased risk of depression in individuals and their spouses.

‡ *p* < 0.01,

† *p* < 0.01.

## Discussion

This study examined the association between the dynamic nature of depression in older couples and the risk of developing possible sarcopenia in a 4-year longitudinal cohort survey. Our study suggested that chronic depression in individuals and their spouses was associated with a significantly increased risk of developing possible sarcopenia compared with a low risk of depression in individuals and their spouses. Depression remission potentially decreased the risk of possible sarcopenia compared to chronic depression. In addition, among individuals without depression, women seemed to be more susceptible to the influence of chronic depression of intimate partners than men, which increased the risk of possible new sarcopenia.

The mechanisms underlying the relationship between depressive symptoms and sarcopenia involved multiple molecule-driven pathways, which included age-related chronic low-grade inflammation, oxidative stress, neurotrophins, and similar lifestyle factors (such as malnutrition and physical inactivity) (Gao K. et al., 2021). Current studies and meta-analyses indicated that sarcopenia is an independent risk factor for depression and depressive symptoms (Chang et al., 2017; Li et al., 2022). However, the opposite direction remains to be explored further, especially in a longitudinal cohort survey. As mentioned earlier, previous cross-sectional studies have reported inconsistent results on depression and the phases of pre-sarcopenia to sarcopenia (Byeon et al., 2016; Szlejf et al., 2019; Endo et al., 2021). A meta-analysis of 11 studies suggested that depression was an independent factor for sarcopenia. However, there were some limitations in this meta-analysis (Gao Q. et al., 2021). All included studies were cross-sectional, and literature retrieval was insufficient. In addition, the diagnosis of depression was based on self-report data in a few studies. Kurita et al. performed a 1-year longitudinal analysis for patients with advanced chronic kidney disease and demonstrated that depression is associated with a higher risk of developing sarcopenia (adjusted OR = 4.64, 95% CI: 1.33–16.2) (Kurita et al., 2021). However, specific individuals and a relatively short follow-up time limited the ability to assess the causal association between depression and sarcopenia. In the study by Kahl et al. (2017) chronic major depressive disorder (MDD) was defined as a depressive symptom without remission for at least 2 years. Acute MDD was defined as a major depressive episode with a duration of <2 years. Chronic MDD was associated with the highest amount of adrenal gland volume, followed by acute MDD and healthy controls. Adrenal gland volume might be considered as a proxy marker for hypercortisolism (Kahl et al., 2017). Hypercortisolism secondary to chronic MDD might play a key role in linking other medical problems (Kahl et al., 2017). Meanwhile, chronic MDD was associated with the dysregulation of hypothalamus pituitary adrenal axis (HPAS), pro-inflammatory cytokines (tumor necrosis factor α (TNF-α) and interleukin-6 (IL-6)), cardiometabolic (blood pressure regulation, glucose, and fat metabolism) systems, etc. (Kahl et al., 2017). In our study, the duration of chronic depression was up to 4 years, and depression remission happened in 2 years without recurrence. This study demonstrated that depression increased the incidence of developing possible sarcopenia in Chinese older adults. The incidence of possible sarcopenia in individuals with depression remission was lower than that in individuals with chronic depression, which indicated the importance of previous depression treatment and the different effects of depression subtype on possible sarcopenia. Compared with previous studies, our 4-year longitudinal cohort study had a higher confidence level in exploring the causal association between depression and sarcopenia.

According to the interdependence theory, the interaction of individuals with intimate relationships might affect emotions, cognition, and outcomes between them (Cook and Kenny, 2015; Marguerite et al., 2017). There was psychological dependence between intimate partners, and the psychological health of one member might affect the health status of another member (Monin et al., 2018). Depression was not only associated with depression and anxiety disorders in intimate partners, but was also associated with partner-reported relationship dysfunction and dissatisfaction (Franz et al., 2020). Depression in intimate partners might increase the risks of cognitive decline and depression in individuals (Monin et al., 2018). Our study firstly observed that chronic depression in intimate partners potentially promoted the incidence of possible sarcopenia in individuals with chronic depression and women who were at low risk of depression. Our study highlighted the interactive consequences of depression among intimate partners and suggested the need for early evaluation and intervention aimed at alleviating both individual-level and couple-level depression. The mechanisms linking adverse outcomes for individuals to depression in intimate partners are not clearly elucidated and remain to be studied further.

Our study also found the sex difference on the association between possible sarcopenia and depression in individuals and their intimate partners who were at low risk of depression. The current study proposed that female individuals are more likely to be affected by the depression symptoms of their spouses (Marguerite et al., 2017). Two studies reported that women are associated with lower quality of life compared to men when facing the mental health of their intimate partners (Maroufizadeh et al., 2018; Pascual-Sáez et al., 2019). One possible explanation is that women are socialized to be interdependent and more sensitive to mental health. In addition, differences in biological susceptibility and genetic and hormonal factors between men and women contributed to the different outcomes. Compared with men, intact women had a greater number of nodes and connections, indicated their intricate molecular response to chronic stress condition (Karisetty et al., 2017). Another sex difference was shown in individuals and their intimate partners with depression remission. After depression remission, the incidence of possible sarcopenia was higher among men than among women. The residual effect of depression on possible sarcopenia seemed to be more obvious in men, even after adjusting for multiple confounding factors.

The main strength of our study was to investigate the effect of a 4-year longitudinal depression trajectory of older adults and their intimate partners on possible sarcopenia using a nationally representative study of the Chinese population. In addition, we also assessed the sex difference among different depression subtypes in terms of developing possible sarcopenia. The main limitation of this study was the lack of some important data of the CHARLS, such as depressive interventions and skeletal muscle mass measured by DXA and BIA, which affected us to better evaluate the association between sarcopenia and depression. Additional limitations included that LCA cannot observe the depression subtype of individuals with depression remission and their intimate partners who were at low risk of depression. In addition, each individual is assigned based on the highest probability of belonging to one of the latent classes, but not actually belonging to a single group in LCA (Mori et al., 2020). Meanwhile, the sample size in the study was relatively small with potential selection bias, especially in classes 3 and 5, which might affect research on the relationship between possible sarcopenia and depression. More studies with large samples are warranted to further explore and test the association between sarcopenia and mental health.

## Conclusions

Our study suggests that the longer the illness duration of depression, the greater a person's risk of developing possible sarcopenia. Our study also highlights the importance of early evaluation for depression in intimate partners. A future large-sample study may benefit from using sarcopenia as measured by DXA and BIA, and examining the association between sarcopenia and an actually single subtype of depression. Health professionals, in their practices, not only screen the risk of depression in individuals to reduce adverse health consequences for older adults, but also highlight the importance of depression in their intimate partners, especially for female individuals.

## Data availability statement

The original contributions presented in the study are included in the article/supplementary material, further inquiries can be directed to the corresponding author/s.

## Ethics statement

Because all related data were derived from the open CHALRS, no patients participated in the recruitment and conduct of the study. This study was deemed exempt from review by the Institutional Review Board at China, Three Gorges University.

## Author contributions

ZH and YT: conceptualization, methodology, investigation, data curation, writing—original draft, and supervision. AY: data curation and investigation. XS: conceptualization and writing—original draft. All authors read and approved the final manuscript.

## Funding

This work was supported by the Behavioral and Social Research division of the National Institute on Aging of the National Institute of Health (Grants Nos. 1-R21-AG031372-01, 1-R01-AG037031-01, and 3-R01AG037031-03S1), the Natural Science Foundation of China (Grants Nos. 70773002, 70910107022, and 71130002), the World Bank (contracts 7145915 and 7159234), and Peking University.

## Conflict of interest

The authors declare that the research was conducted in the absence of any commercial or financial relationships that could be construed as a potential conflict of interest.

## Publisher's note

All claims expressed in this article are solely those of the authors and do not necessarily represent those of their affiliated organizations, or those of the publisher, the editors and the reviewers. Any product that may be evaluated in this article, or claim that may be made by its manufacturer, is not guaranteed or endorsed by the publisher.

## References

[B1] Alexopoulos G. S. (2019). Mechanisms and treatment of late-life depression. Transl. Psychiatry 9, 188. 10.1038/s41398-019-0514-6 31383842 PMC6683149

[B2] Byeon C. H. Kang K. Y. Kang S. H. Kim H. K. Bae E. J. (2016). Sarcopenia is not associated with depression in Korean adults: results from the 2010–2011 Korean national health and nutrition examination survey. Korean J. Fam. Med. 37, 37–43. 10.4082/kjfm.2016.37.1.37 26885321 PMC4754285

[B3] Chang K. V. Hsu T. H. Wu W. T. Huang K. C. Han D. S. (2017). Is sarcopenia associated with depression? A systematic review and meta-analysis of observational studies. Age Ageing 46, 738–746. 10.1093/ageing/afx094 28633395

[B4] Chen H. Xiong P. Chen L. Hao G. (2020). Childhood neighborhood quality, friendship, and risk of depressive symptoms in adults: the China health and retirement longitudinal study. J. Affect. Disord. 276, 732–737. 10.1016/j.jad.2020.07.090 32736183

[B5] Chen L. K. Woo J. Assantachai P. Auyeung T. W. Chou M. Y. Iijima K..H.. (2020). Asian working group for Sarcopenia: 2019 consensus update on sarcopenia diagnosis and treatment. J. Am. Med. Dir. Assoc. 21, 300–307. 10.1016/j.jamda.2019.12.012 32033882

[B6] Cheng H. G. Chen S. McBride O. Phillips M. R. (2016). Prospective relationship of depressive symptoms, drinking, and tobacco smoking among middle-aged and elderly community-dwelling adults: results from the China health and retirement longitudinal study (CHARLS). J. Affect. Disord. 195, 136–143. 10.1016/j.jad.2016.02.023 26895091

[B7] Cipolli G. C. Aprahamian I. Borim F. Falcão D. Cachioni M. Melo R. C.. (2021). Probable sarcopenia is associated with cognitive impairment among community-dwelling older adults: results from the FIBRA study. Arquivos de neuro-psiquiatria 79, 376–383. 10.1590/0004-282X-ANP-2020-0186 34161525 PMC9394561

[B8] Cook W. Kenny D. (2015). The actor–partner interdependence model: a model of bidirectional effects in developmental studies. Int. J. Behav. Dev. 29, 101–109. 10.1080/01650250444000405

[B9] Cruz-Jentoft A. J. Sayer A. A. (2019). Sarcopenia. Lancet 393, 2636–2646. 10.1016/S0140-6736(19)31138-9 31171417

[B10] Delibaş D. H. Eşkut N. Ilhan B. Erdogan E. Top Karti D. Yilmaz Küsbeci Ö.. (2021). Clarifying the relationship between sarcopenia and depression in geriatric outpatients. Aging Male 24, 29–36. 10.1080/13685538.2021.1936482 34151708

[B11] Endo T. Akai K. Kijima T. Kitahara S. Abe T. Takeda M.. (2021). An association analysis between hypertension, dementia, and depression and the phases of pre-sarcopenia to sarcopenia: a cross-sectional analysis. PLoS ONE 16, e0252784. 10.1371/journal.pone.0252784 34292967 PMC8297796

[B12] Franz M. R. Kaiser A. P. Phillips R. J. Lee L. O. Lawrence A. E. Taft C. T.. (2020). Associations of warzone veteran mental health with partner mental health and family functioning: family foundations study. Depress. Anxiety 37, 1068–1078. 10.1002/da.23083 32805764 PMC8252135

[B13] Gao K. Ma W. Z. Huck S. Li B. L. Zhang L. Zhu J.. (2021). Association between sarcopenia and depressive symptoms in chinese older adults: evidence from the China health and retirement longitudinal study. Front. Med. 8, 755705. 10.3389/fmed.2021.755705 34869454 PMC8635632

[B14] Gao Q. Hu K. Yan C. Zhao B. Mei F. Chen F.. (2021). Associated factors of sarcopenia in community-dwelling older adults: a systematic review and meta-analysis. Nutrients 13, 4291. 10.3390/nu13124291 34959843 PMC8707132

[B15] Hu Y. Peng W. Ren R. Wang Y. Wang G. (2022). Sarcopenia and mild cognitive impairment among elderly adults: the first longitudinal evidence from CHARLS. J. Cachexia Sarcopenia Muscle. 10.1002/jcsm.13081 36058563 PMC9745544

[B16] Hu Z. Song X. Hu K. Ruan Y. Zeng F. (2021). Association between sleep duration and asthma in different weight statuses (CHNS 2009–2015). Sleep Breath. Schlaf Atmung 25, 493–502. 10.1007/s11325-020-02081-6 32335852

[B17] Hu Z. Tian Y. Song X. Zeng F. Yang A. (2022). Associations between sarcopenia with asthmatic prevalence, lung function and comorbidity. BMC Geriat. 22, 703. 10.1186/s12877-022-03394-9 36002808 PMC9404581

[B18] Jiang C. H. Zhu F. Qin T. T. (2020). Relationships between chronic diseases and depression among middle-aged and elderly people in China: a prospective study from CHARLS. Curr. Med. Sci. 40, 858–870. 10.1007/s11596-020-2270-5 33123901

[B19] Kahl K. G. Herrmann J. Stubbs B. Krüger T. H. Cordes J. Deuschle M.. (2017). Pericardial adipose tissue and the metabolic syndrome is increased in patients with chronic major depressive disorder compared to acute depression and controls. Prog. Neuropsychopharmacol. 72, 30–35. 10.1016/j.pnpbp.2016.08.005 27528109

[B20] Karisetty B. C. Khandelwal N. Kumar A. Chakravarty S. (2017). Sex difference in mouse hypothalamic transcriptome profile in stress-induced depression model. Biochem. Biophys. Res. Commun. 486, 1122–1128. 10.1016/j.bbrc.2017.04.005 28385526

[B21] Kim M. Sasai H. Kojima N. Kim H. (2015). Objectively measured night-to-night sleep variations are associated with body composition in very elderly women. J. Sleep Res. 24, 639–647. 10.1111/jsr.12326 26250860

[B22] Kristensen M. T. Hulsbæk S. Faber L. L. Kronborg L. (2021). Knee extension strength measures indicating probable sarcopenia is associated with health-related outcomes and a strong predictor of 1-year mortality in patients following hip fracture surgery. Geriatrics 6, 8. 10.3390/geriatrics6010008 33467771 PMC7839049

[B23] KuritaN.WakitaT.FujimotoS.YanagiM.KoitabashiK.SuzukiT.. (2021). Hopelessness and depression predict sarcopenia in advanced CKD and Dialysis: a multicenter cohort study. J. Nutr. Health Aging 25, 593–599. 10.1007/s12603-020-1556-433949624

[B24] Li Q. Xu Y. Zhou H. Loke A. Y. (2016). Factors influencing the health-related quality of life of Chinese advanced cancer patients and their spousal caregivers: a cross-sectional study. BMC Palliat. Care 15, 72. 10.1186/s12904-016-0142-3 27484209 PMC4971682

[B25] Li Z. Tong X. Ma Y. Bao T. Yue J. (2022). Prevalence of depression in patients with sarcopenia and correlation between the two diseases: systematic review and meta-analysis. J. Cachexia Sarcopenia Muscle 13, 128–144. 10.1002/jcsm.12908 34997702 PMC8818614

[B26] Lim S. K. Kong S. (2022). Prevalence, physical characteristics, and fall risk in older adults with and without possible sarcopenia. Aging Clin. Exp. Res. 10, 100. 10.1007/s40520-022-02078-z 35133613

[B27] Maeda K. Akagi J. (2017). Cognitive impairment is independently associated with definitive and possible sarcopenia in hospitalized older adults: the prevalence and impact of comorbidities. Geriatr. Gerontol. Int. 17, 1048–1056. 10.1111/ggi.12825 27273820

[B28] Malhi G. S. Mann J. J. (2018). Depression. Lancet 392, 2299–2312. 10.1016/S0140-6736(18)31948-2 30396512

[B29] Marguerite S. Laurent B. Marine A. Tanguy L. Karine B. Pascal A.. (2017). Actor-partner interdependence analysis in depressed patient-caregiver dyads: influence of emotional intelligence and coping strategies on anxiety and depression. Psychiatry Res. 258, 396–401. 10.1016/j.psychres.2017.08.082 28890228

[B30] Maroufizadeh S. Hosseini M. Rahimi Foroushani A. Omani-Samani R. Amini P. (2018). The effect of depression on quality of life in infertile couples: an actor-partner interdependence model approach. Health Qual. Life Outcom. 16, 73. 10.1186/s12955-018-0904-0 29690877 PMC5937824

[B31] Monin J. K. Doyle M. Van Ness P. H. Schulz R. Marottoli R. A. Birditt K.. (2018). Longitudinal associations between cognitive functioning and depressive symptoms among older adult spouses in the cardiovascular health study. Am. J. Geriatr Psychiatry 26, 1036–1046. 10.1016/j.jagp.2018.06.010 30120019 PMC6280660

[B32] Mori M. Krumholz H. M. Allore H. G. (2020). Using latent class analysis to identify hidden clinical phenotypes. JAMA 324, 700–701. 10.1001/jama.2020.2278 32808993

[B33] Pascual-Sáez M. Cantarero-Prieto D. Blázquez-Fernández C. (2019). Partner's depression and quality of life among older Europeans. Eur. J. Health Econ. 20, 1093–1101. 10.1007/s10198-019-01081-y 31218579

[B34] Ruiz M. Hu Y. Martikainen P. Bobak M. (2019). Life course socioeconomic position and incidence of mid-late life depression in China and England: a comparative analysis of CHARLS and ELSA. J. Epidemiol. Community Health 73, 817–824. 10.1136/jech-2019-212216 31255999

[B35] Sinha P. Calfee C. S. Delucchi K. L. (2021). Practitioner's guide to latent class analysis: methodological considerations and common pitfalls. Crit. Care Med. 49, e63–e79. 10.1097/CCM.0000000000004710 33165028 PMC7746621

[B36] Szlejf C. Suemoto C. K. Brunoni A. R. Viana M. C. Moreno A. B. Matos S.. (2019). Depression is associated with sarcopenia due to low muscle strength: results from the ELSA-Brasil study. J. Am. Med. Dir. Assoc. 20, 1641–1646. 10.1016/j.jamda.2018.09.020 30409492

[B37] Valiengo L. Stella F. Forlenza O. V. (2016). Mood disorders in the elderly: prevalence, functional impact, and management challenges. Neuropsychiatr. Dis. Treat. 12, 2105–2114. 10.2147/NDT.S94643 27601905 PMC5003566

[B38] Wei Y. Wang Z. Wang H. Li Y. Jiang Z. (2019). Predicting population age structures of China, India, and Vietnam by 2030 based on compositional data. PLoS ONE 14, e0212772. 10.1371/journal.pone.0212772 30973941 PMC6459537

[B39] Zhao Y. Hu Y. Smith J. P. Strauss J. Yang G. (2014). Cohort profile: the China health and retirement longitudinal study (CHARLS). Int. J. Epidemiol. 43, 61–68. 10.1093/ije/dys203 23243115 PMC3937970

